# Asap: A Framework for Over-Representation Statistics for Transcription Factor Binding Sites

**DOI:** 10.1371/journal.pone.0001623

**Published:** 2008-02-20

**Authors:** Troels T. Marstrand, Jes Frellsen, Ida Moltke, Martin Thiim, Eivind Valen, Dorota Retelska, Anders Krogh

**Affiliations:** 1 Bioinformatics Centre, Department of Molecular Biology and Biotech Research and Innovation Centre (BRIC), University of Copenhagen, Copenhagen, Denmark; 2 Swiss Institute of Bioinformatics, Swiss Institute for Experimental Cancer Research (ISREC), Epalinges, Switzerland; Wellcome Trust Centre for Human Genetics, United Kingdom

## Abstract

**Background:**

In studies of gene regulation the efficient computational detection of over-represented transcription factor binding sites is an increasingly important aspect. Several published methods can be used for testing whether a set of hypothesised co-regulated genes share a common regulatory regime based on the occurrence of the modelled transcription factor binding sites. However there is little or no information available for guiding the end users choice of method. Furthermore it would be necessary to obtain several different software programs from various sources to make a well-founded choice.

**Methodology:**

We introduce a software package, Asap, for fast searching with position weight matrices that include several standard methods for assessing over-representation. We have compared the ability of these methods to detect over-represented transcription factor binding sites in artificial promoter sequences. Controlling all aspects of our input data we are able to identify the optimal statistics across multiple threshold values and for sequence sets containing different distributions of transcription factor binding sites.

**Conclusions:**

We show that our implementation is significantly faster than more naïve scanning algorithms when searching with many weight matrices in large sequence sets. When comparing the various statistics, we show that those based on binomial over-representation and Fisher's exact test performs almost equally good and better than the others. An online server is available at http://servers.binf.ku.dk/asap/.

## Introduction

Efficient identification of transcription factor binding sites is a crucial initial step in the study of gene regulation. We are often interested in identifying over-represented transcription factor binding sites (TFBSs) in some set of hypothesised co-regulated genes as this indicates that the set share a common regulatory mechanism. Modelling the binding of transacting proteins to cis-regulatory sequences by computational approaches is becoming increasingly important in hypothesis testing and generation.

The binding preference of a known transcription factor can be described by the sequences to which it binds. Aligning the sequences and counting the nucleotides at each position in the alignment provides a *count matrix* similar to those found in databases as TRANSFAC [1] and JASPAR [2]. Log-transforming this *count matrix*, taking into account the background nucleotide distribution of the genomic region of interest, provides the position weight matrix (PWM). Various algorithms can then be used to scan a set of sequence with this PWM to identify likely binding sites. However, due to the short and degenerate nature of TFBSs a typical PWM will detect a hit every 500–5000 base-pair depending on parameter settings [3]; leading to a genome-wide number of predictions that are much higher than estimates from experimental data [4].Two different approaches are frequently employed to decrease the large number of presumably false positives. One is phylogenetic footprinting where conservation of the detected sites in orthologous promoters are used as evidence for functionality, see the review in [5] and examples of tools in [6]–[8]. A disadvantage of this method is its inability to detect species-specific regulatory mechanisms and the sensitivity to the alignment of the regulatory regions. The other approach is to ignore the mapping of the specific binding sites and calculate an over-representation statistic for the transcription factor to assess whether it is the likely cause of the observed co-regulation. Here we focus on a handful of methods for assessing over-representation.

The assumption behind an over-representation statistics is that functional TFBSs will be over-represented in the set of co-regulated genes as compared to a background set [9] (by the term co-regulated we refer to a set of genes hypothesized to be co-regulated either based on expression data or some other information). Several methods exist for assessing the significance of over-representation [10]–[13], but most of these methods are implemented in distinct tools for promoter analysis making a comparison between the different statistics cumbersome. However, these methods all rely on some comparison of the distribution of TFBSs, modelled by PWMs, between two sequence sets and they can therefore be implemented in a common framework. We here present such an implementation: A fast search algorithm coupled with an easily extendable framework for calculating the different test-statistics.

When interested in finding a common regulatory regime for a set of co-regulated genes, the main objective is to find the representative regulators, whereas the mapping of their actual binding sites in the DNA sequences as a secondary objective that may require different statistics. Our goal is to systematically test various parameters on diverse but controlled sequence sets in order to establish a guideline for conducting optimal promoter analysis. In doing so, we focus on the hypothesis testing capability of the statistics rather than their ability to map the location of the actual TFBSs.

An important caveat of this entire framework is that even if a TFBS is significantly over-represented it does not imply biological function directly as several epigenetic features may further modulate the transcriptional events [14]–[16].

## Materials and Methods

Computational identification of transcription factor binding sites consists of two parts: scoring and assessment. We will deal with each in turn.

### Scoring

Scoring is done using a PWM representing a specific TFBS. To take into account the base composition of the promoters a background model from a relevant set of sequences is estimated. The background model is usually a Markov model representing either the relative frequencies of the nucleotides A, C, G, and T (zeroth order); the 16 di-nucleotides (first order) or any word-length of nucleotides (n-th order). Often there is too little information in the original alignments to estimate anything but a zeroth order model for the transcription factor binding site, however it can be combined with a higher order background model to take into account dependencies in the nucleotide composition. Effectively the PWM is the log ratio of the conditional pattern probabilities and conditional background probabilities (see supplementary material, text S1)

Having defined the PWM it becomes a matter of finding all sub-sequences of length W (the width of the PWM) scoring above a given threshold. These sub-sequences are considered the predicted binding sites for the transcription factor in question. If the sequence sets (positive and background set) are large, or if we wish to search with several PWMs, this can be a computationally taxing problem. We have implemented a C library using a data structure called enhanced suffix arrays (ESA). Using a modified version of the ESAsearch algorithm, introduced by Beckstette et al. [17], we are able to solve the scoring problem with a speedup of as much as a factor 1000 compared to a naïve implementation (see supplementary material, text S1). The primary benefit of ESAsearch is that whenever two or more W-sub-sequences share a prefix the score for that prefix is only calculated once. Additionally, a look-ahead principle is used: The scoring of any given sub-sequence is stopped if the intermediate score of any of its prefixes plus the highest possible score for the rest of the sub-sequence is below the threshold. Combining these two principles are especially advantageous; when the scoring of a sub-sequence is stopped due to the look-ahead principle, ESAsearch also discards all other sub-sequences that share the prefix that led to the stop.

Further speedup is achieved by utilizing the fact that TFBSs, and thus PWMs, are short. We can use this to impose an upper bound on the prefixes (currently set to 50) which efficiently speeds up the sorting when building the ESA by a factor two compared to the sophisticated *lcp* algorithm by T. Kasai et al. [18] (see supplementary material, text S1).

A disadvantage of using an ESA is that the data structure uses nine times as much memory as the size of the input sequences. Since the building time is linear in the size of the input sequences, it is only advantageous when searching the sequence set with multiple PWMs (see table 1 and supplementary material, text S1, for speed comparisons).

**Table 1:** Speed comparison to naïve search

File size	Our ESAsearch	Naïve	Searches
**36 MB**	0.20	2.44	15
**8 MB**	0.13	1.22	14
**4 MB**	0.04	0.27	12
**1 MB**	0.01	0.07	8

Search time for our implementation compared to a naïve search. The final column indicates the number of PWMs to search with to ‘break-even’ with the naïve search taking into account the building time of the enhanced suffix array

### Assessment

The strength of over-representation can be expressed by a test-statistic. Here we have implemented and rigorously tested the performance of several published methods that are used within the field: The binomial over-representation used by TOUCAN [12], the Fisher's exact test and z-score used by oPOSSUM [10], [11], the area under the ROC used by Clarke et al [10], the log-ranking employed by PAP [13], and finally the Wilcoxon rank sum test. We include the Wilcoxon rank sum as it represents a formalized statistic in the same genre as those employed by [10] and [13]. To the best of our knowledge no current tool uses Wilcoxon rank sum test for assessment of over-represented TFBSs.

As the statistics are sensitive to the sequence lengths we concatenate the background sequences *after* searching for matches and then partition the concatenated sequences into sequences of equal length – the mean length of the positive sequences. By concatenating after all instances have been found, we avoid forming ‘new’ words in the boundaries of the sequences.

Finally our statistics module is interfaced to R [19] using Rpy. This enables the user to take advantage of the rich statistical framework provided by R and easily extend the currently implemented methods.

## Results

We test all implemented statistics on an artificial data set, somewhat similar to Tompa et al. [20], in order to control all variables. Originally these methods where tested on diverse data sets and a direct comparison based on the original literature is therefore impossible. However, we do acknowledge that our artificial data set may indeed promote some statistics compared to others. E.g. the ranking statistics, area under ROC and ln-rank, both rely on a sum of PWM scores within each sequence. Thus these statistics would benefit from several TFBSs in each positive sequence, and here we only place one. As we are aware that the artificial data set may not fully represent the complex structure of a true biological data set we also assess the different statistics on a ChIP data set from Wei et al. [21].

### Data

Our data set consists of 117 positive sequence sets from dbTSS [22], each with a total 100 sequences. Each sequence in a specific sequence set have a probability of having an embedded site from a specific JASPAR CORE 2008 PWM [23]. The probability is 100% for the performance test on the order of the background model, 50% for the tests of statistics across multiple thresholds and finally between 10–90% in the dilution test. Our background set consists of 1000 sequences also from dbTSS. For testing the speed of the implemented search algorithm we choose a set of ∼31000 dbTSS sequences.

The data from Wei et al. [21] consists of DNA fragments from a p53 ChIP experiment that are converted into pair-ended di-tags (PETs) and mapped back to the human genome. Here we use all 323 PET tag clusters with 3 or more counts as our positive set.

### Speed test

To test the speed of our implemented algorithm we partition the master file (the 31000 sequences) into several smaller ones. Using 50 randomly chosen PWMs with a threshold giving an expected match every 10000 base pair we compare our implementation to a naïve search. The results are given in table 1. The last column indicates the number of PWMs one would need to search with in order to ‘break-even’ with the naïve method when taking into account the building time of the enhanced suffix array, (see supplementary material, text S1, for a more detailed comparison). All tests were done on a 2.4 GHz Intel Pentium 4 processor with 1.5 GB of memory running Linux. We used the sum of user and sys times as reported by the Linux time command.

### Background model order

**Figure 1:**
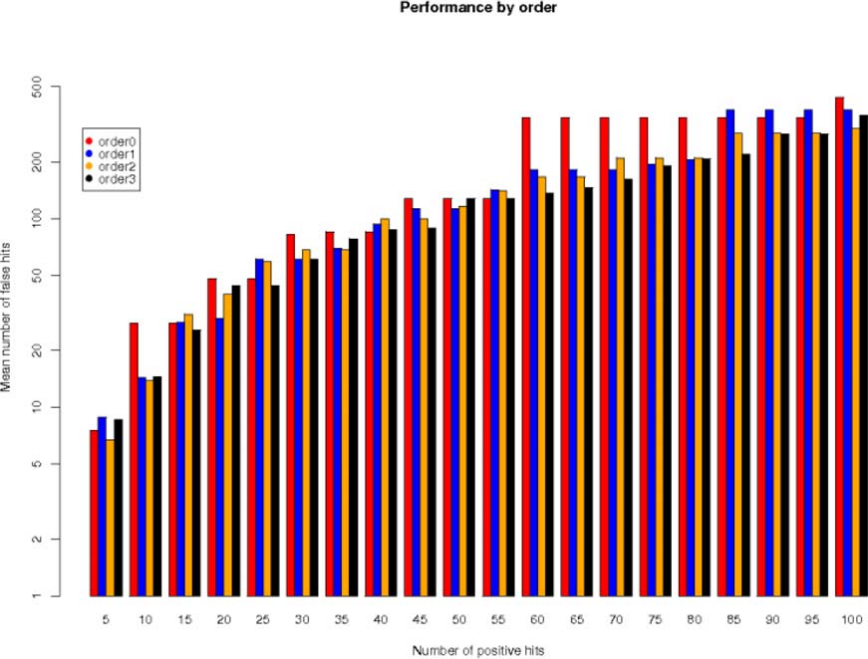
Performance of PWMs based on background model. Average number of false hits in the background sequences per hit in the positive sequences across 117 JASPAR CORE PWMs.

It has been shown in [24] that a high-order Markov chain is a better background model than the standard zeroth order. To find the appropriate order we scan all data sets with the respective PWM and record the number of true instances found in the positive sequences (all sequences have an embedded site) and the mean number of instances found in the background set. Figure 1 shows a small increase in performance by order, and we decided to continue comparing order 0 and order 3.

Based on this we test all statistics with a sequence set with 50% chance of an embedded site with both zeroth order and third order background models. For each positive data set we calculate all over-representation statistics for all matrices and record if the true matrix was found (the one corresponding to the embedded sites) and the number of possibly false matrices, that is, other matrices also showing significant over-representation in the set. Thus we have an overall number of 117 true predictions and (117×138)–117 = 16029 possible false predictions. We use a p-value threshold of 0.05, a z-score threshold of 3, an area under the ROC above 0.5, and ln-rank score above 2 as suggested by the original papers. We do not correct for multiple testing. Results are summarized in table 2.

**Table 2:** Comparison of over-representation statistics based on background model.

Order 0	Binomial	Z-score	Fisher's	ROC	Wilcoxon	Ln-rank
TRUE	99	67	95	54	21	53
FALSE	1046	4073	539	10386	2871	2326
Ppv.	0.0865	0.016	0.150	0.005	0.007	0.022
Sens.	0.846	0.573	0.812	0.462	0.180	0.453
FPR	0.065	0.254	0.034	0.648	0.180	0.145
Spec.	0.935	0.746	0.966	0.352	0.821	0.855
**Order 3**	**Binomial**	**Z-score**	**Fisher**'**s**	**ROC**	**Wilcoxon**	**Ln-rank**
TRUE	92	59	87	59	26	48
FALSE	1522	3878	1219	5387	5785	2035
Ppv.	0.057	0.015	0.067	0.011	0.004	0.023
Sens.	0.786	0.504	0.744	0.504	0.222	0.410
FPR	0.095	0.242	0.076	0.336	0.361	0.127
Spec.	0.905	0.758	0.924	0.664	0.639	0.873

Performance of the different over-representation statistics based on a zeroth and third order background model. The PWM threshold is 0.9 of the scoring range.

The trend (previously observed in [24]) of higher background giving higher performance is not present in our test. In fact only the poorly performing statistics seems to borrow strength from the higher order background, while the better performing statistics are hurt by the increase in background model. Thus we select zeroth order background models to further boost the better performing statistics. Also when testing across a series of thresholds (0.9, 0.8, 0.7, and 0.6) of each PWM specific scoring range ((max−min)*threshold+min) it is clear that the optimal statistic is the Fishers exact test, data not shown. Finally, in the dilution test it is evident that this statistic is also relatively robust with respect to the number of sites in the positive set never dropping below a sensitivity of 50% as shown in table 3.

**Table 3:** Dilution test using Fisher's exact test.

Prob.	10%	20%	30%	40%	50%	60%	70%	80%	90%
TRUE	61	75	85	87	95	97	97	102	102
FALSE	395	433	465	492	539	573	604	652	681
Sens.	0.521	0.641	0.726	0.744	0.812	0.829	0.829	0.872	0.872
Spec.	0.975	0.973	0.971	0.969	0.966	0.964	0.962	0.960	0.958

Sensitivity and specificity measures based on the probability of embedded JASPAR sites across all 138 PWMs and 117 sequence sets, no correction for multiple testing.

### ChIP data

We partition the data from Wei et al. [21] into four groups based on the number of counts in the PET tag cluster. The first data set consists of all sequences with six counts, the next of all sequences with five or more counts, etc until all 323 sequences with 3 or more counts are included. Thus we successively weaken the p53 signal. For each of the four data sets we search with all the JASPAR 2008 CORE PWMs using a loose threshold of 0.8 of the maximum scoring range. We then rank the significance values from each statistic and record the rank of the PWM for p53, see table 4. As other transcription factors may be present in the positive set our major concern is the statistics ability to specify p53 as being the most significantly over-represented feature. The results correspond with our results obtained on the artificial data sets: the best performing statistics is the binomial over-representation and Fisher's exact test.

**Table 4:** Rank of the p53 PWM on ChIP data

PET count	Binomial	Z-score	Fisher's	ROC	Wilcoxon	Ln-rank
**6**	1*	1*	1*	94	25*	1*
**5**	1*	1*	1*	79	97	1*
**4**	1*	1*	1*	73.5	137	1*
**3**	1*	62	8*	1*	36.5	1*

The rank of the PWM for p53 using the different statistics, * indicates that the significance value provided is significant at the 0.05 level.

## Discussion

The apparently contradictive result that the zeroth order PWM performs better than the third order highlights some of the problems of over-representation statistics, or more generally PWM scoring. Confounding factors are numerous and include: the threshold value, PWM to PWM similarity, and the information content of the PWM.

Firstly, calculating the threshold of the PWM based on the scoring range of the model it is clear that including a higher order background model will effectively lead to an altered scoring range and thus affect the absolute threshold value. In our specific case this affects the performance differences of over-representation statistics between the zeroth order and third order PWMs. Secondly, since transcription factors of similar function sometimes bind to similar sequence patterns they are not independent. In other words, if PWM *A* is very similar to PWM *B* both of them will likely be deemed significantly over-represented in the sequences with the embedded *A* sites and vice versa. Thirdly comparing performance across a set of different PWMs all with different information content is difficult. Obviously different information content leads to different binding affinities and how to interpret the p-values derived from low and high information content PWMs is not trivial. All these confounding effects influence the final value calculated by the over-representation statistics and influence our ability to compare the values obtained by different PWMs.

In reality the problem of promoter analysis is further complicated by different promoter architectures [25], and therefore sub-partitioning the sequences and background models as suggested by Down and Hubbard [26] would be justified. However, this further limits the ability to compare the resulting over-representation without expert biological knowledge. Furthermore we have, in the current work, not considered the effect of overlapping and/or palindromic sites. Such sites will clearly affect the resulting test-statistics. However, further analyses are required to quantify the effects and find solutions to handle such sites intelligently.

Despite the severe difficulties related to promoter analysis in mammalian genomes, our analysis shows that over-represented transcription factors are detectable using current methods even for low sites to sequences ratios.

As for the program package it can be easily extended to include various other types of genomic data. An obvious extension would be to include conservation tracks and other data tracks from the UCSC genome browser in a coherent manner.

Here we focus on the usage of the program package within the field of promoter analysis, however, all patterns that can be represented by a PWM can potentially benefit from our framework. Our current implementation provides the community with a basic framework for fast searching with PWMs and integrated analyses of the results either through the current implemented methods or by use of the rich statistical framework provided by R. Finally our framework can be use directly from our web interface at: http://servers.binf.ku.dk/asap/

## Supporting Information

Text S1 Higher order background models and detailed speed comparison.
(0.07 MB PDF)
