# Supplementary material for: Asap: A Framework for Over-Representation Statistics for Transcription Factor Binding Sites
Source: PLoS One. 2008 Feb 20;3(2):e1623. doi: 10.1371/journal.pone.0001623 (PMC2229843; doi:10.1371/journal.pone.0001623)
Supplement: Text S1 — Higher order background models and detailed speed comparison. (0.07 MB PDF) [file pone.0001623.s001.pdf]

Asap: A framework for over-representation statistics for transcription factor binding sites, supplementary material

1 High-order models

Having both a motif model and a background model we can define the PWM as the log-ratio between the motif frequency and the corresponding background frequency across all nucleotides and all positions. Assume that the motif model is an order m

W

File size	File type	copy	cache	qsufsort	cr	ds	our ds
36 MB	single	33.94	46.40				

File size	
